# Kaposi′s Sarcoma and Acute Limb Ischaemia: A Possible Direct Vascular Link

**DOI:** 10.1155/crom/3340869

**Published:** 2026-06-26

**Authors:** Berthe Sabine Mapoko Esson, Etienne Okobalemba Atenguena, Lionel Fossa Tabola, Rachel Tayou, Dominique Ndom Anaba, Anne Sango, Paul Ndom

**Affiliations:** ^1^ Department of Internal Medicine and Specialties, University of Yaounde I, Yaounde, Cameroon, uy1.uninet.cm; ^2^ Department of Internal Medicine, University of Dschang, Dschang, Cameroon, univ-dschang.org; ^3^ Department of Clinical Sciences, University of Buea, Buea, Cameroon, ubuea.cm

**Keywords:** acute limb ischaemia, gangrene, Kaposi′s sarcoma

## Abstract

Acute limb ischaemia is caused by a sudden interruption in arterial flow. Its occurrence in oncology is uncommon compared with the general population. Generally considered to be part of the paraneoplastic syndrome, its occurrence in a patient with Kaposi′s sarcoma (KS) raises the question of a different pathophysiology. We report a case of acute limb ischaemia in a patient with KS. The patient was 51 years old and had hyperpigmented macules on both lower limbs for 6 months, some of which had become nodules. He was recently diagnosed with HIV and had been put on treatment. He presented with purplish macules on the lower limbs, and examination of the oral cavity revealed a purplish lesion on the palate. He presented with dry gangrene of the left limb that worsened over time. A vascular link could be suggested as an explanation for acute limb ischaemia in KS.

## 1. Introduction

Acute limb ischaemia (ALI) is caused by a sudden interruption in arterial flow. It threatens the viability of the limb and is considered acute if it lasts less than 2 weeks [1]. Its occurrence in oncology is uncommon compared with the general population [2]. There are many possible causes, including embolic etiologies and microcirculatory damage associated with connective tissue diseases or paraneoplastic syndromes. ALI may precede, coincide with or follow the diagnosis of cancer. It is generally considered to be part of the paraneoplastic syndrome. Its occurrence in a patient with Kaposi′s sarcoma (KS), a tumour that develops at the expense of endothelial cells, raises the question of a different pathophysiology. We report a case of ALI in a patient with KS, while asking whether there might be a direct vascular link between the two conditions.

## 2. Case Presentation

This was a 51‐year‐old patient who had been presenting with hyperpigmented macules on both lower limbs for 6 months, some of which had become nodules. These dermatological lesions were accompanied by intermittent pain of mild to moderate intensity. He also complained of chronic diarrhoea for 2 months, with liquid stools that were not bloody. A progressively disabling physical asthenia had set in. He had used traditional medicines for a month without any improvement. He did not have claudication. He was recently diagnosed with human immunodeficiency virus (HIV) and had been put on treatment. His CD4 count was 120 cells/mm^3^. His triple therapy consisted of dolutegravir 50, lamivudine 300 and tenofovir 300. He was not diabetic. He consumed alcohol but not tobacco.

**Figure 1:**
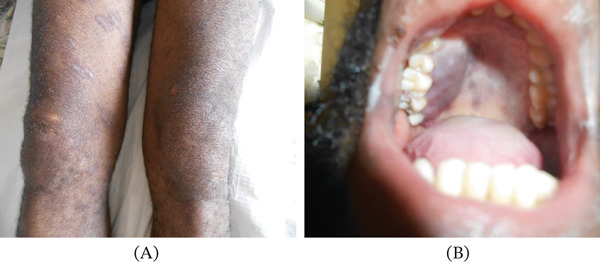
(A) Purplish macules on both lower limbs; (B) purplish lesion on the palate.

**Figure 2:**
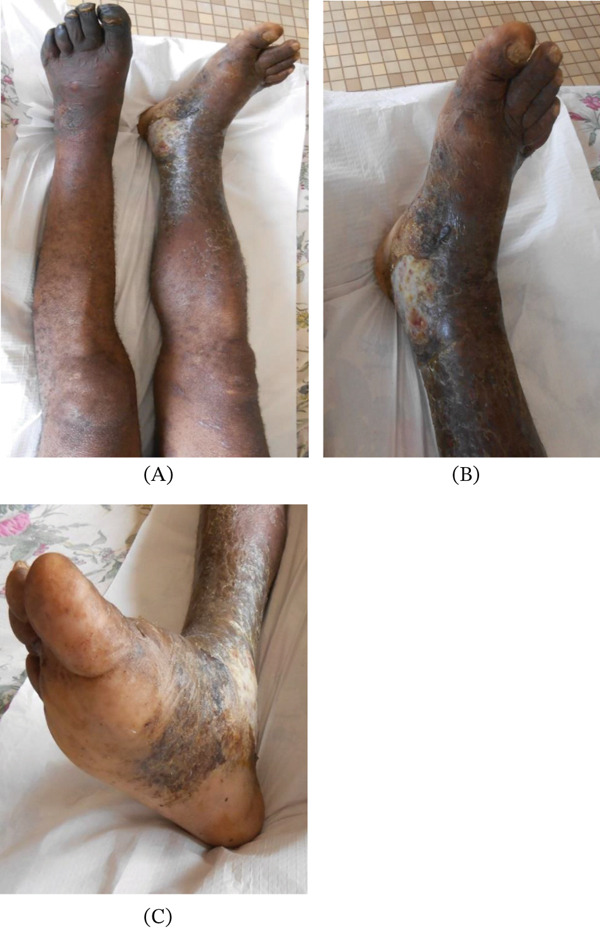
(A) Lymphoedema of the right lower limb; (B, C) crusts associated with necrosis and desquamation of the skin over the medial malleolus of the right foot.

**Figure 3:**
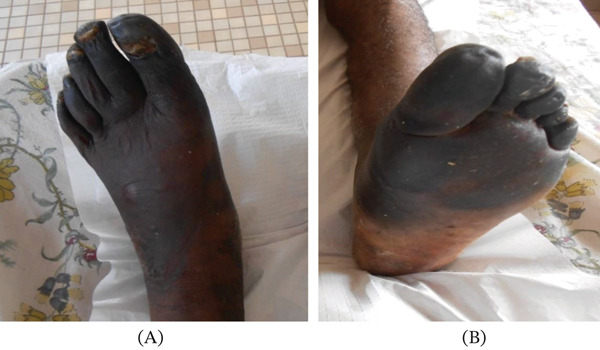
Dry gangrene of the left foot: (A) dorsal surface and (B) plantar surface.

On physical examination, his general condition was altered, with a WHO Stage 3 performance index. He had purplish macules on his lower limbs (Figure 1A). On the right lower limb, he had lymphedema, crusts associated with necrosis and scaling of the skin around the medial malleolus (Figure 2). The left foot was black (since 02 weeks before the hospitalisation) and cold (Figure 3). He had dry gangrene, which worsened during hospitalisation. Examination of the oral cavity revealed a purplish lesion on the palate (Figure 1B). Abdominal, cardiac and pulmonary examinations revealed no abnormalities.

### 2.1. Diagnostic Management

A biopsy of the purplish lesions and an angioscan of the lower limbs were recommended. The patient was unable to undergo both. The chest x‐ray was normal, and the transthoracic cardiac ultrasound showed moderate precapillary pulmonary hypertension and a left ventricular ejection fraction of 65%. The blood count showed haemoglobin 13.7 g/L, leucocytes 12970/mm^3^, neutrophils 11480/mm^3^, platelets 304.103/mm^3^ and urea/creatinine levels of 2.66 g/L and 12.7 mg/L, respectively.

### 2.2. Treatment

Antibiotic therapy was administered for 5 days. The patient underwent two haemodialysis sessions. The acute kidney injury identified during the investigation raised concerns about the presence of tenofovir in the patient′s antiretroviral regimen. It should be removed from the regimen and replaced by a molecule that does not impair kidney function. A leg amputation was proposed for dry gangrene of the left foot. The patient was subsequently to receive chemotherapy for KS. The planned protocol was weekly paclitaxel.

Follow‐up and outcome of care correction of renal function was achieved after haemodialysis sessions. The inflammatory syndrome was controlled. The patient was discharged against medical advice and was lost to follow‐up without treatment of the dry gangrene and KS being initiated.

## 3. Discussion

The incidence of ALI is approximately 2/100,000 per year [3]. ALI is rare, but its incidence is increasing, suggesting that it may be part of a paraneoplastic syndrome [4]. Little is known about the pathophysiology of this phenomenon. There are several hypotheses, most of which tend to explain it as a paraneoplastic syndrome. Vascular spasm, overproduction of vasoconstrictor factors, intimal proliferation, intraluminal thrombosis, vasculitis, an immune complex or drug toxicity are the main hypothetical factors leading to ALI [4]. ALI has been described in cancers of various organs: lung, breast, colon, stomach, oesophagus, cervix, larynx, melanoma and lymphoma [4]. Our patient had KS. Although the patient did not undergo biopsy, the lesions he presented were typical of KS. He had purplish macules on the skin and a purplish lesion on the palate. Certain clinical situations could mimic KS, such as bacillary angiomatosis, pyogenic granuloma or cutaneous vasculitis [5]. In bacillary angiomatosis, the patient may have multiple papules and nodules as the lesions often arise in crops, and the usual site is the upper extremities [6]. Furthermore, lymphoedema is not among the possible skin lesions described for bacillary angiomatosis [7]. Nodular KS usually shows numerous dome‐shaped nodular lesions, unlike pyogenic granuloma, which often presents with a single lesion [8]. Cutaneous vasculitis described are nonnodular lesions that are not associated with intraoral lesions [9]. In our patient, the presence of an intraoral nodular lesion rules out this diagnosis. In the literature, we found one case of ALI described in a patient with KS [10]. We believe that ours is the second case of ALI described in a patient with KS. Although the patient was unable to undergo vascular imaging of the limbs, he had several thrombotic risk factors which taken together could lead to an ALI. He had HIV, KS and was highly immunosuppressed with a CD4 count of 120 cells/mm^3^. Each of these three factors has been described as contributing to thrombosis. The pathophysiology of ALI in our KS patient may not be the paraneoplastic syndrome that is generally postulated. As KS affects the blood vessels, and ALI is due to a sudden interruption in arterial flow, a direct vascular link could be suggested. KS is a multicentric malignant tumour arising from endothelial cells in blood vessels, characterised by clinical heterogeneity depending on the immune status of the patient [11]. KS is caused by human herpesvirus 8 (HHV8), also known as Kaposi′s sarcoma–associated herpesvirus (KSHV) [12]. It is responsible for endothelial proliferation, with inflammatory infiltrates and vascular leakage [12]. This local inflammation and proliferation could lead to vascular occlusion. Several viruses such as the HIV and herpes simplex virus have been implicated in the pathogenesis of vasculitis in variable vessel sizes [13]. Patients presenting with ALI from thrombosis usually present with worsening claudication symptoms and rest pain [14]. It was not the case with our patient, thereby ruling out a thrombotic cause for his gangrene. The most common source for an embolus (a cause of ALI) is the heart [14]. Since the patient′s cardiac examination revealed no abnormalities, a cardiac cause for an embolism would be unlikely. In addition, infections, occlusive disease due to a hypercoagulable state and vasculitis are some of the mechanisms suggested for gangrene of the extremities in HIV [15]. Viral infections have been implicated in the pathogenesis of systemic vasculitis, and many viruses, including HIV, are associated with vasculitis and occasionally gangrene [15].

The two major mechanisms by which infection is thought to induce a vasculitis are direct microbial invasion, with resultant damage of the vessel wall, and immune‐mediated injury, both humoral and cellular [16].

Viral‐induced endothelial injury causes increased levels of von Willebrand factor, total antigenic protein S, plasminogen activator inhibitor (PAI‐1), endothelial‐derived thrombomodulin and other procoagulant products of endothelial cell activation [17].

There is evidence that in situ thrombosis, as a result of active vasculitis, can lead to ischaemia and gangrene, as seen in angiographic examination of affected patients, as well as on histological examination of affected tissue showing arterial thrombi [18]. As these phenomena occur in a vessel affected by KS, a direct vascular link could be evoked to explain ALI in a patient with KS.

## 4. Limitations

Since a biopsy and histopathological analysis were not performed, certain differential diagnoses for the final diagnosis of KS could have been considered. The specific clinical features that ruled out these differential diagnoses were identified in order to confirm the diagnosis of KS.

## 5. Conclusion

Although ALI is considered to be part of the paraneoplastic syndrome in oncology, it could hypothetically also be caused by direct vascular damage in patients with KS.

## Author Contributions

Decision to submit the manuscript for publication: L.F.T. Manuscript drafting: L.F.T., E.O.A., B.S.M.E., and R.T. Manuscript revision: D.N.A., A.S., and P.N. All listed authors made significant contributions.

## Funding

No funding was received for this manuscript.

## Disclosure

All authors read and approved the final manuscript, and all of them agree to be accountable for the research presented. The authors take full responsibility for the content of the publication.

## Consent

No written consent has been obtained from the patients as there is no patient identifiable data included in this case report.

## Conflicts of Interest

The authors declare no conflicts of interest.

## Data Availability

The data that support the findings of this study are available on request from the corresponding author. The data are not publicly available due to privacy or ethical restrictions.
